# Exaggerated perception of facial expressions is increased in individuals with schizotypal traits

**DOI:** 10.1038/srep11795

**Published:** 2015-07-02

**Authors:** Shota Uono, Wataru Sato, Motomi Toichi

**Affiliations:** 1 Department of Neurodevelopmental Psychiatry, Habilitation and Rehabilitation, Faculty of Human Health Science, Graduate School of Medicine, Kyoto University, 53 Shogoin Kawahara-cho, Sakyo-ku, Kyoto 606-8507, Japan; 2 Faculty of Human Health Science, Graduate School of Medicine, Kyoto University, 53 Shogoin Kawahara-cho, Sakyo-ku, Kyoto 606-8507, Japan; 3 The Organization for Promoting Developmental Disorder Research, 40 Shogoin Sannou-cho, Sakyo-ku, Kyoto 606-8392, Japan

## Abstract

Emotional facial expressions are indispensable communicative tools, and social interactions involving facial expressions are impaired in some psychiatric disorders. Recent studies revealed that the perception of dynamic facial expressions was exaggerated in normal participants, and this exaggerated perception is weakened in autism spectrum disorder (ASD). Based on the notion that ASD and schizophrenia spectrum disorder are at two extremes of the continuum with respect to social impairment, we hypothesized that schizophrenic characteristics would strengthen the exaggerated perception of dynamic facial expressions. To test this hypothesis, we investigated the relationship between the perception of facial expressions and schizotypal traits in a normal population. We presented dynamic and static facial expressions, and asked participants to change an emotional face display to match the perceived final image. The presence of schizotypal traits was positively correlated with the degree of exaggeration for dynamic, as well as static, facial expressions. Among its subscales, the paranoia trait was positively correlated with the exaggerated perception of facial expressions. These results suggest that schizotypal traits, specifically the tendency to over-attribute mental states to others, exaggerate the perception of emotional facial expressions.

Facial expressions indicate emotional states and communicative intentions^1^ and hence are indispensable for human social interactions. To enhance cooperation and reduce struggles, we must detect other’s facial expressions rapidly and to appropriately respond to them. The ability to recognize facial expressions also has a long-lasting effect. Previous studies showed that people who are sensitive to other’s facial expressions achieve high social status^2^, are successful in business^3^, and experience high subjective well-being and low depression^4^. Thus, it is important to understand what characteristics moderate an individual’s ability to process another’s facial expression.

Previous studies demonstrated that some psychiatric characteristics are associated with impaired facial expression processing, and are correlated with their daily social function^5^,^6^. A recent study revealed that psychiatric characteristics could modulate perception, which is an early stage in the processing of others’ facial expressions^7^. The researchers investigated the perception of dynamic and static facial expressions in individuals with autism spectrum disorder (ASD) and typically developing controls. ASD is characterized by qualitative impairments in social interaction^8^; individuals with ASD have difficulty recognizing others’ facial expressions^9^ and do not spontaneously attribute mental states to others^10^. The study presented dynamic and static facial expressions and asked participants to change an emotional face display to match the perceived final image from dynamic and static facial expression stimuli. Typically developing controls and individuals with ASD were likely to perceive the presented facial expressions as exaggerated in general^7^,^11^,^12^. They also perceived the final image of dynamic facial expressions as more exaggerated than static facial expressions. Compared to the controls, the degree of exaggeration was reduced in individuals with ASD when the intensity of dynamic facial expressions was subtle. Further, the degree of reduced perception of dynamic facial expressions was related to social dysfunction severity. These results suggest that the exaggerated perception of dynamic facial expressions is reduced as a function of autistic traits. The results further suggest the possibility that other types of psychiatric characteristics related to different types of social impairment may correlate with the exaggerated perception of dynamic facial expressions in different ways.

We hypothesized that the degree to which certain characteristics were present on the schizophrenia spectrum disorder, including schizophrenia and schizotypal personality disorder^8^, may be associated with exaggerated perception of dynamic facial expressions. The characteristics of these disorders are observed to a lesser degree in the general population^13^. Schizophrenia spectrum disorder is characterized by the pervasive impairment of social cognition, as shown in ASD, such as theory of mind^14^ and emotion recognition^15^. However, it has been proposed that, unlike ASD, individuals with these disorders tend to excessively attribute mental states due to dysfunctional social cognition^16^,^17^. While individuals with high autistic traits were less likely to perceive agency in humans, those with high schizotypal traits inappropriately attributed agency and experience to inanimate objects^18^. Other behavioral studies have demonstrated that individuals with schizophrenia and high schizotypal traits interpret sentences and actions as more intentional compared to controls^19^,^20^. These findings suggest that different psychological mechanisms may underlie the social cognitive dysfunctions that characterize autism and schizophrenia, and schizophrenic characteristics have an opposite effect than ASD on the perception of dynamic facial expressions.

Recent studies investigating face processing have demonstrated certain biases in line with the finding that individuals with schizophrenic characteristics tend to excessively attribute mental states. The studies have demonstrated that individuals with schizophrenia perceive the gaze direction of others in a self-referential manner; i.e., they have a greater tendency to perceive an averted gaze as a direct gaze compared with controls^21^,^22^. In terms of facial expression recognition, it has been reported that individuals with schizophrenia and high schizotypal traits are likely to categorize neutral facial expressions as fearful or angry^23^,^24^,^25^. The existence of these biases suggests that individuals with schizophrenic characteristics, specifically the tendency to excessively attribute mental states, also exhibit a strong perceptual bias concerning the facial expressions of others. We hypothesized that the exaggerated perception of dynamic facial expressions would increase with schizophrenic characteristics. Furthermore, based on a theoretical suggestion^16^,^17^, we hypothesized that the exaggerated perception of facial expressions would be specifically related to the tendency to over-attribute mental states to others in individuals with schizophrenic characteristics.

To test these hypotheses, we investigated the relationship between exaggerated perception of dynamic facial expressions and schizophrenic characteristics in a normal population. To test perception of facial expressions, we used the same experimental paradigm used to investigate perception of facial expressions in ASD^7^. We presented dynamic and static facial expressions at subtle, medium, and extreme intensities, and asked participants to change an emotional face display to match the perceived final image from dynamic and static facial expression stimuli. To investigate the effect of schizophrenic characteristics, we measured schizotypal traits in the general population because: (1) non-clinical individuals with high schizotypal traits have similar cognitive performance as patients with schizophrenia, independent of general intellectual ability^26^; and (2) it allowed us to investigate the effect of schizophrenic characteristics without the possible confounding effect of medication and poor motivation. After the experiment, participants completed the Schizotypal Personality Questionnaire^27^ (SPQ), which measures various aspects of schizophrenic characteristics. We predicted that individuals with high schizotypal traits would perceive dynamic facial expressions as more exaggerated than did those with few schizotypal traits.

We further analyzed the relationships between the exaggerated perception of dynamic facial expressions and the schizotypal trait subscales to test our second hypothesis. Two SPQ subscales are highly relevant to the tendency to overestimate another’s mental state: (1) “suspiciousness” is the tendency to suspect to negative and harmful intentions of others, and (2) “ideas of reference” is the feeling of being subjected by others and external events, and it is an underlying characteristic of suspiciousness^28^. Recent studies showed that these symptoms construct a single factor of “paranoia”^29^,^30^. Based on these data, we predicted that individuals with high paranoia would perceive dynamic facial expressions as more exaggerated.

## Methods

### Participants

Forty-six undergraduate and graduate students at Kyoto University participated in this study (26 males; mean ± standard deviation [SD]: age, 21.22 ± 1.73 years). One participant (female) was excluded from the analysis because of her ophthalmologic problem. All other participants had normal or corrected-to-normal visual acuity. The participants provided written informed consent and the study was approved by the Institutional Ethics Committee. The study was also conducted in accord with the Declaration of Helsinki.

### Design

The experiment was constructed as a two-factorial design, with presentation condition (dynamic or static) and intensity (52%, 80%, or 108%) as the repeated factors.

### Stimuli

Dynamic and static facial expression stimuli were identical to those used in a previous study^7^. From a set of facial images^31^, we selected one neutral expression slide and two emotional expression (fearful and happy) slides for each of four actors (two males and two females). Using computer-morphing techniques, intermediate images between the neutral expressions (0%) and each of the full emotional expressions (100%) were created in 4% steps. For dynamic facial expression stimuli, face images from 4% to a maximum of 52%, 80%, or 108% of the original emotional expression were sequentially presented in 4% steps. To create the 108% emotional expression images, the facial features of the 100% emotional expression were changed in the direction opposite from that depicted in the neutral face. The 52%, 80%, and 108% conditions consisted of 13, 20, and 27 image frames in succession, respectively. The total presentation times were 130 ms, 200 ms, and 270 ms for the 52%, 80%, and 108% conditions, respectively. Under the static condition, the last frame of each dynamic facial expression stimulus was used. The presentation time was the same as that for the dynamic facial expression with the corresponding intensity.

### Apparatus

Events were controlled using a program written in Visual C++ 5.0 (Microsoft) on a Windows computer (HP xw4300 Workstation). Stimuli were presented on a 17-inch CRT monitor (Iiyama; refresh rate 100 Hz). The distance between the monitor and participants was fixed at approximately 57 cm using a headrest.

### Procedure

The procedure was identical to that used in a previous study^7^. Two windows were bilaterally presented on the monitor. The vertical and horizontal visual angles of these windows were 11.1 and 7.8 degrees, respectively. First, a crosshair was presented at the center of a stimulus window (i.e., left window). The participants were instructed to fixate on this. Then, a dynamic or static facial expression was presented in the window, and 250 ms later, a face image of the same person was presented in a response window (i.e., right window). Participants were asked to match the image in the response window exactly with the last image shown as the dynamic or static facial expression in the stimulus window by using the mouse to drag a slider to the left or right. The face shown as the initial image in the response window had an emotional expression of −10, 0, or +10% intensity of the presented stimuli. The upper or lower limit of the slider scale had one of three predefined ranges, which covered an 80% intensity range (e.g., under the 52% condition, 2–82, 12–92, or 22–102%). The scale ranges varied randomly across trials and were invisible to the participants. After a participant selected an image, he or she clicked a button, and the image in the response window disappeared. Then, the stimulus was presented again in the stimulus window, and 250 ms later, the image chosen by the participant appeared in the response window. If the participant thought the images matched exactly, he or she clicked the button and went on to the next trial; if not, the participant could modify the image. No time limits were set for the two judgments. Each participant completed several practice trials to familiarize themselves with face image manipulation using the mouse. A total of 48 trials (eight trials per condition) were included in blocks, and the trial order was counterbalanced across participants.

### Questionnaire

To assess schizotypal traits, participants were asked to complete the Japanese version of the SPQ^32^ after the experiment. The SPQ is a self-report measure consisting of 74 items in which participants answer if they agree or disagree with the statements (e.g., “Do you sometimes feel that other people are watching you?”). The response format is “yes/no.” Nine subscales exist within the SPQ: ideas of reference, odd beliefs and magical thinking, unusual perceptual experience, suspiciousness, excessive social anxiety, no close friends, constricted affect, odd and eccentric behavior, and odd speech. For the Japanese version of the SPQ, Cronbach’s alpha of subscales (α > 0.76) was the same degree as the original version, except that those of the odd belief and magical thinking and unusual perceptual experience subscales were relatively low. We assigned one point to a yes response for each item, and calculated the total SPQ score and the score of each SPQ subscale. The means (with SD) of subscales, factors, and total score of the SPQ are shown in Table 1. The total SPQ score in the present study (mean ± SD, 24.9 ± 11.0) was similar with that of a previous large-sample study in Japan^26^ (mean ± SD, 25.1 ± 12.0).

### Data analysis

For each participant, the mean intensity of response images across emotion categories was calculated for each condition; preliminary analyses showed that the relationship between SPQ scores and behavioral measures did not differ according to emotion category (see Supplement 1 for details). Then, the ratio between response intensity and presented images was calculated for each condition. The ratios were subjected to a 2 (presentation) × 3 (intensity) repeated-measures analysis of variance (ANOVA). To confirm that dynamic facial expressions were perceived in a more exaggerated form than were static expressions, a simple main-effect analysis was conducted for each intensity condition.

To analyze the relationships between exaggerated perception and schizotypal traits, Pearson’s product–moment correlations were calculated between the performance and the SPQ score. As the factorial analysis did not reveal any interaction between intensity and presentation condition, we averaged across intensity conditions. We analyzed the data under both dynamic and static presentation conditions, although we did not have specific predictions about the effect of static presentations. According to a previous study^30^, we used the SPQ total score and the composite scores reflecting four factors: (1) positive symptoms (odd belief and magical thinking, and unusual perceptual experiences), (2) negative symptoms (excessive social anxiety, no close friends, constricted affect, and suspiciousness), (3) disorganized symptoms (odd and eccentric behavior, and odd speech), and (4) paranoia (ideas of reference and suspiciousness) (see Table 1). The significance of correlation coefficients was evaluated using *t*-tests (two-tailed).

We explored whether the data were normally distributed for each behavioral measure (dynamic and static condition) and questionnaire score (positive, negative and disorganized symptoms, paranoia and total SPQ score) using Kolmogorov-Smirnov tests. The results showed that no measures, with the exception of positive symptoms, violated the assumption of normal distribution (*p* > 0.10). Furthermore, the results of non-parametric and parametric tests of the relationship between behavioral measures and positive symptoms were similar. Therefore, we reported only the results of the parametric tests.

## Results

### The perception of dynamic and static facial expressions

**Figure 1:**
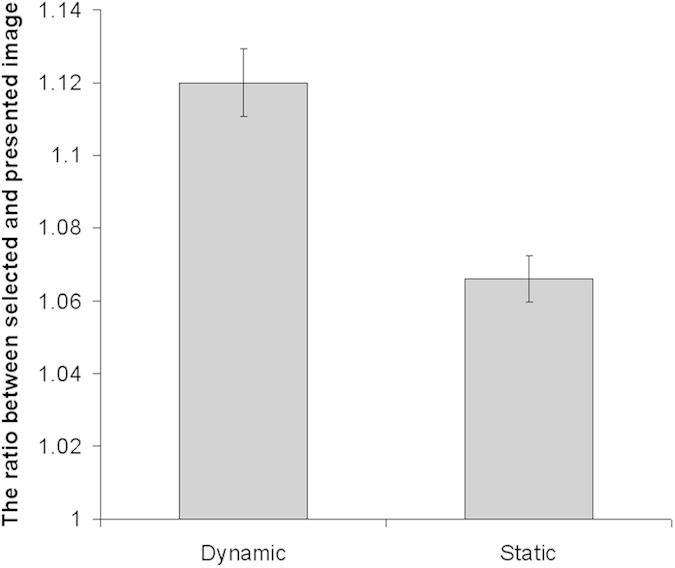
Mean ratio between the intensity of the selected and presented images for the dynamic and static conditions. Error bars represent the standard errors (SE).

First, exaggeration of facial expressions reported in previous studies^7^,^11^,^12^ was investigated. The mean response under each condition (with standard error, *SE*) is shown in Table 2. The ratios between the intensity of response images and presented images were calculated and subjected to a presentation × intensity ANOVA. The results revealed a main effect of presentation (*F*[1, 44] = 74.90, *p* < 0.001), indicating that participants perceived more exaggerated images under dynamic than under static conditions (Fig. 1). A main effect of intensity was also found (*F*[2, 88] = 41.07, *p* < 0.001). Although the interaction was not significant (*F*[2, 88] = 1.90, *p* > 0.10), we conducted a simple effect analyses and confirmed that dynamic facial expressions were perceived as more emotionally intense than static facial expressions under each intensity condition (all *p* < 0.05).

### Relationship between the exaggerated perception of facial expressions and schizotypal traits

Then, the relationships between the exaggerated perception of facial expressions and schizotypal traits were investigated. The correlational analyses results are shown in Table 3. We found a significant positive correlation between total SPQ score and exaggerated perception of dynamic facial expressions (*r*[45] = 0.364, *p* = 0.014), indicating that people with high schizotypal traits perceived dynamic facial expressions as more exaggerated than those with few schizotypal traits. When we analyzed the static condition data, a significant positive correlation was also found between the exaggerated perception of static facial expressions and the total SPQ score (*r*[45] = 0.328, *p* = 0.028).

**Figure 2:**
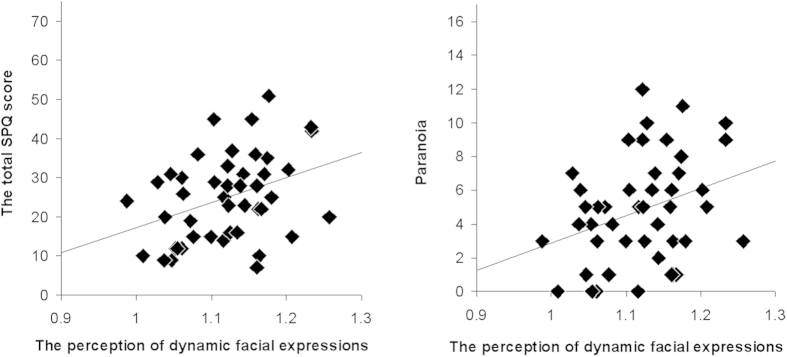


Finally, the relationships between the exaggerated perception of facial expressions and composite schizotypal trait scores (Table 3) were investigated. For the exaggerated perception of dynamic facial expressions, significant positive correlations were found with paranoia (*r*[45] = 0.320, *p* = 0.032) (Fig. 2), as well as negative symptoms (*r*[45] = 0.379, *p* = 0.010). For the static facial expressions, the same correlational relationships were found to be significant (paranoia: *r*[45] = 0.337, *p* = 0.024; negative symptoms: *r*[45] = 0.319, *p* = 0.033). The significant correlations between negative symptoms and exaggerated perception may be due to the fact that this latter construct included the suspiciousness subscale. When the suspiciousness scores were excluded from the overall negative symptoms score, the correlation with exaggerated perception, of dynamic but not static facial expressions, remained significant (dynamic: *r*[45] = 0.358, *p* = 0.016; static: *r*[45] = 0.244, *p* > 0.10).

## Discussion

Our results showed that participants perceived the final dynamic facial expression images to be more exaggerated than the static facial expressions. The perceived emotional intensity of dynamic and static facial expressions emerged in all intensity conditions. The results replicated previous findings in typically developing individuals and those with ASD^7^,^11^,^12^.

More importantly, our results demonstrated that individuals who scored high on schizotypal traits perceived dynamic facial expressions as more exaggerated than those who had few schizotypal traits. These results are consistent with our hypothesis that the exaggerated perception of dynamic facial expressions would increase in accordance with the degree of schizophrenic characteristics. Interestingly, our results also showed that static facial expressions were perceived as more exaggerated in individuals with high schizotypal traits. Our additional analysis revealed a significant correlation between performance in the dynamic and static conditions (*r*[45] = 0.747, *p* < 0.001), suggesting that exaggerated perception is a general phenomenon that is not restricted to dynamic facial expression perception, and furthermore that participants respond to dynamic and static facial expressions in a consistent fashion. A previous study reported that static and dynamic facial expression recognition is moderated by schizotypal traits^33^,^34^. However, no study tested the effect of schizotypal traits on the perception of facial expressions. To our knowledge, this is the first evidence indicating that individuals with high schizotypal traits generally perceive others’ emotional facial expressions in an exaggerated manner.

Our results further revealed that, among the sub-components of schizotypal traits, paranoia, which consists of the suspiciousness and ideas of reference scales, was related to the exaggerated perception of facial expressions. Suspiciousness is the tendency to suspect negative and harmful intentions of others. Ideas of reference is the feeling of being subjected by others and external events. Items on these two scales indicate the tendency to erroneously detect attention and intention from others. Thus, individuals with a high paranoia score are likely to over-attribute mental states to others. In fact, previous studies demonstrated that individuals with high schizotypal traits^20^ and paranoid schizophrenia^35^ interpret the movement of biological or non-biological objects based on the intentional stance. Together with these findings, our results suggest that the tendency to over-attribute mental states to others in individuals with high schizotypal traits is associated with exaggeration of the perception of others’ facial expressions.

Interestingly, the present finding that individuals with high schizotypal traits perceive others’ emotional facial expressions in an exaggerated manner contrasts with a previous finding in ASD^7^. The study showed that the ASD group perceived a reduced degree of exaggeration for dynamic facial expressions under the subtle intensity condition. Further, the exaggerated perception of subtle dynamic facial expressions negatively correlated with social dysfunction severity. Although both individuals with ASD and schizophrenia exhibit impaired social cognition^36^, it is possible that the underlying psychological mechanisms related to the social cognition impairments differ between ASD and schizophrenia^16^,^17^. The lack of spontaneous mental state attribution to others would cause social cognition dysfunction in individuals with ASD, while the tendency to over-attribute mental states to others might contribute to the impairments in those with schizophrenia. Consistent with this proposition, the opposite relationship between autistic and schizotypal traits in the exaggerated perception of facial expressions suggests that ASD and schizophrenia are at two extremes of the continuum with respect to social cognitive abilities, such as the attribution of mental state.

One might argue that increased exaggeration of perception of facial expression along with schizotypal traits contradicts the presence of emotion recognition deficits in individuals with schizophrenia. A meta-analysis revealed that patients with schizophrenia are impaired in terms of recognizing emotional facial expressions irrespective of task type^15^. Recent studies extended the research into the general population, and showed that individuals with schizotypal traits are less accurate at recognizing others’ emotions from their faces^34^,^37^. However, some studies revealed that a subgroup of schizophrenia had an emotion recognition performance distinct from that of other groups. One study found that the subgroup with mild social perception impairment included more persons with paranoid schizophrenia than that of the severe impairment group^38^. Another study demonstrated that individuals with paranoid schizophrenia were accurate at recognizing genuine but not posed facial expressions compared with other schizophrenic groups and controls^39^. Based on the possibility that exaggerated perception of facial expressions is useful to effectively detect others’ emotions, the current study suggests that high paranoia (e.g., ideas of reference and suspiciousness) has a positive effect on detecting and recognizing emotion from others’ faces.

It is possible that the exaggerated perception of facial expressions impairs social interactions. In daily communication, facial expressions are ambiguous and facial movements do not necessarily relate to the expresser’s emotion. Based on the present results, individuals with high schizotypal traits may perceive clear facial expressions even from ambiguous expressions and unrelated facial movements and hence attribute incorrect emotions to others. In fact, previous studies demonstrated that individuals with schizophrenia and high schizotypal traits are likely to categorize neutral faces as angry or fearful^23^,^24^,^25^, particularly during ongoing paranoid symptoms^40^. Given that neutral faces may be judged as expressing negative emotions, such as anger, even in typical individuals^41^,^42^, the exaggerated perception of facial expressions in individuals with high schizotypal traits may further contribute to the misattribution of emotion in others’ faces. Consistent with this notion, individuals with schizophrenia and persecutory delusions are more likely to perceive hostility from others^43^,^44^. Promising directions for further research include investigating whether the exaggerated perception of facial expressions (specifically anger) in individuals with high schizotypal traits (e.g., ideas of reference and suspiciousness) deteriorate their daily social function.

In addition to paranoia, negative symptoms were positively correlated with exaggerated perception of dynamic and static facial expressions. Even after exclusion of the suspiciousness score, the correlation with exaggerated perception of dynamic, but not static, facial expressions remained significant. This suggests that negative symptoms also contribute to the exaggerated perception of dynamic facial expressions. However, in several studies certain of these symptoms and characteristics (e.g., social anxiety and no close friends) were included in the paranoia factor, although such studies have also consistently considered ideas of reference and suspiciousness as indicative of paranoia^45^,^46^. Based on these findings, paranoia and certain negative symptoms may share an underlying psychological process and increase the propensity toward exaggerated perception of facial expressions.

Some limitations of the current study should be noted. First, we used only non-clinical participants. Although it is assumed that schizophrenic traits constitute a spectrum in the general population^13^, further studies are needed to investigate whether the results of the present study can be extended to patients with schizophrenia. Further, direct comparisons between individuals with schizophrenia and ASD would allow us to test the hypothesis that ASD and schizophrenia are at two extremes of the continuum in terms of the ability of mental state attribution. Second, we did not measure intellectual ability, because university students do not appear to be characterized by general intellectual impairment; moreover, the current task did not require verbal responses. Although there is controversy regarding which aspects of intellectual ability are associated with schizophrenic characteristics^47^, disentangling the effects of schizophrenia characteristics and intelligence quotient (IQ) represents an important topic for future research. Finally, our sample size was relatively small. Post hoc power analyses for significant correlations revealed a power range of between 0.597 and 0.766. Several of the current results (specifically the non-significant correlations), may not be amenable to replication in studies using large samples. Further investigation using such samples is required to validate the characteristics of each schizophrenia trait.

## Conclusions

In summary, the present study showed that participants perceived the final dynamic facial expression images to be more exaggerated than static facial expressions. Further, participants with high schizotypal traits perceived more exaggerated dynamic and static facial expressions than those with few schizotypal traits. The paranoia and negative symptoms scores were also positively correlated with exaggerated perception of dynamic and static facial expressions. This suggests that schizotypal traits, particularly the tendency to over-attribute mental states to others, is related to the exaggerated perception of facial expressions.

## Additional Information

**How to cite this article**: Uono, S. *et al.* Exaggerated perception of facial expressions is increased in individuals with schizotypal traits. *Sci. Rep.* **5**, 11795; doi: 10.1038/srep11795 (2015).

## Supplementary Material

Supplementary Information

## Figures and Tables

**Table 1:** Mean (*SD*) scores of the SPQ subscales.

**Subscales**		**Mean (SD)**
1.	Ideas of reference	1.98 (1.66)
2.	Odd belief and magical thinking	0.80 (1.12)
3.	Unusual perceptual experience	1.91 (1.76)
4.	Suspiciousness	2.84 (2.02)
5.	Excessive social anxiety	5.04 (2.02)
6.	No close friend	2.89 (2.25)
7.	Constricted affect	2.89 (2.26)
8.	Odd and eccentric behavior	2.07 (1.97)
9.	Odd speech	4.49 (2.36)
Factors		
	Positive symptom (2, 3)	2.71 (2.38)
	Negative symptom (4, 5, 6, 7)	13.67 (6.64)
	Disorganized symptom (8, 9)	6.56 (3.56)
	Paranoia (1, 4)	4.82 (3.18)
The total score		
	The SPQ	24.9 (11.0)

SD: standard deviation; SPQ: Schizotypal Personality Questionnaire.

**Table 2:** Mean (with *SE*) intensities of the selected images.

Presentation	**Intensity**		
	52%	80%	108%
Dynamic	60.4 (0.7)	90.0 (0.9)	115.9 (1.0)
Static	56.9 (0.6)	85.8 (0.7)	111.4 (0.7)

**Table 3:** The correlations between the perception of facial expression and the SPQ.

	**Positive**	**Negative**	**Disorganized**	**Paranoia**	**Total**
Dynamic	0.105	0.379*	0.219	0.320*	0.364*
Static	0.068	0.319*	0.292	0.337*	0.328*

^*^p < .05.
