# Supplementary material for: Exaggerated perception of facial expressions is increased in individuals with schizotypal traits
Source: Sci Rep. 2015 Jul 2;5:11795. doi: 10.1038/srep11795 (PMC4488831; doi:10.1038/srep11795)
Supplement: Supplementary Information [file srep11795-s1.doc]

**Running Head**: Facial expression perception and schizotypy

# Exaggerated perception of facial expressions is increased in individuals with schizotypal traits

# Shota Uono1,, Wataru Sato1,, Motomi Toichi2,3

# Department of Neurodevelopmental Psychiatry, Habilitation and Rehabilitation, Faculty of Human Health Science, Graduate School of Medicine, Kyoto University, 53 Shogoin Kawahara-cho, Sakyo-ku, Kyoto 606-8507, Japan.

# Faculty of Human Health Science, Graduate School of Medicine, Kyoto University, 53 Shogoin Kawahara-cho, Sakyo-ku, Kyoto 606-8507, Japan.

# The Organization of Promoting Developmental Disorder Research, 40 Shogoin Sannou-cho, Sakyo-ku, Kyoto 606-8392, Japan.

# Correspondence concerning this article should be addressed to Shota Uono, Department of Neurodevelopmental Psychiatry, Habilitation and Rehabilitation, Faculty of Human Health Science, Graduate School of Medicine, Kyoto University, 53 Shogoin Kawahara-cho, Sakyo-ku, Kyoto 606-8507, Japan. Tel: +81- 75-751-3966; Fax: +81-75-751-3966; E-mail: uonoshota1982@gmail.com

**Supplement 1.**

We conducted a preliminary 2 (presentation) × 2 (emotion) x 3 (intensity) analysis of variance and found a significant interaction between emotion category and presentation (*F*(1,44) = 6.41, *p* = 0.02). Post hoc analysis revealed that the perception of fearful expressions tended to be exaggerated compared to happy expressions in the dynamic (*F*(1,44) = 3.11, *p* = 0.08), but not in the static (*F*(1,44) = 1.27, *p* = 0.27), condition. This suggests that dynamic presentations exert a greater influence on the perception of fearful facial expressions compared to happy expressions. However, when the emotion category was included as a within-participant factor, each condition comprised only four trials. Furthermore, when we conducted a 2 (presentation) × 2 (emotion) ANCOVA, using each SPQ scale as a covariate, there were no significant interactions, including between SPQ scale scores and emotion (*F*s (1,43) < 1.668, *p*s > 0.20). Finally, we choose to collapse this factor
